# Self-Powered and Autonomous Vibrational Wake-Up System Based on Triboelectric Nanogenerators and MEMS Switch

**DOI:** 10.3390/s22103752

**Published:** 2022-05-14

**Authors:** Yuan Lin, Youchao Qi, Jiaqi Wang, Guoxu Liu, Zhaozheng Wang, Junqing Zhao, Yi Lv, Zhi Zhang, Ning Tian, Mengbi Wang, Yuanfen Chen, Chi Zhang

**Affiliations:** 1 School of Mechanical Engineering, Guangxi University, Nanning 530004, China; 1911301021@st.gxu.edu.cn; 2 CAS Center for Excellence in Nanoscience, Beijing Key Laboratory of Micro-Nano Energy and Sensor, Beijing Institute of Nanoenergy and Nanosystems, Chinese Academy of Sciences, Beijing 100083, China; qiyouchaoo@163.com (Y.Q.); 202010410039@imut.edu.cn (J.W.); liuguoxu@binn.cas.cn (G.L.); wangzhaozheng@binn.cas.cn (Z.W.); zhaojunqing@binn.cas.cn (J.Z.); lvyi@binn.cas.cn (Y.L.); zhangzhi@binn.cas.cn (Z.Z.); 3 School of Nanoscience and Technology, University of Chinese Academy of Sciences, Beijing 100049, China; 4 Tsinghua Innovation Center in Zhuhai, Zhuhai 519080, China; tianning@tsinghua-zh.cn; 5 State Key Laboratory of Precision Measurement Technology and Instruments Department of Precision Instrument Tsinghua University, Beijing 100084, China; wmb@mail.tsinghua.edu.cn; 6 Center on Nanoenergy Research, School of Physical Science and Technology, Guangxi University, Nanning 530004, China

**Keywords:** triboelectric nanogenerator, vibrational energy harvesting, self-powered accelerometer, wake-up system, MEMS switch, autonomous wireless sensing

## Abstract

With the extensive application of wireless sensing nodes, the demand for sustainable energy in unattended environments is increasing. Here, we report a self-powered and autonomous vibrational wake-up system (SAVWS) based on triboelectric nanogenerators and micro-electromechanical system (MEMS) switches. The energy triboelectric nanogenerator (E-TENG) harvests vibration energy to power the wireless transmitter through a MEMS switch. The signal triboelectric nanogenerator (S-TENG) controls the state of the MEMS switch as a self-powered accelerometer and shows good linearity in the acceleration range of 1–4.5 m/s^2^ at 30 Hz with a sensitivity of about 14.6 V/(m/s^2^). When the acceleration increases, the S-TENG turns on the MEMS switch, and the wireless transmitter transmits an alarm signal with the energy from E-TENG, using only 0.64 mJ. Using TENGs simultaneously as an energy source and a sensor, the SAVWS provides a self-powered vibration monitoring solution for unattended environments and shows extensive applications and great promise in smart factories, autonomous driving, and the Internet of Things.

## 1. Introduction

With the development of the Internet of Things (IoT), wireless sensor nodes (WSN) have been widely applied to environmental monitoring and data analysis [1]. The huge number of nodes and broad distribution cause great challenges to the sustainable energy supply of wireless nodes [2]. Traditional WSNs are powered by batteries [3], which has the associated problems of battery replacement and environmental pollution, especially in harsh environments and remote unattended areas. Harvesting mechanical energy from the environment is a significant method for realizing energy conservation, environmental protection, and self-powered wireless devices. As a new energy harvesting technology, the triboelectric nanogenerator (TENG) invented in 2012 has shown excellent advantages, such as its lightweight, simple structure, and extensive material [4,5,6,7]. In recent years, the TENG has been proven that can be used to harvest various environmental mechanical energies, such as wind energy [8,9], ocean energy [10,11], vibrational energy [12,13], and human kinetic energy [14,15]. However, the low efficiency of energy conversion limits the application of the TENG. To improve TENG energy conversion efficiency, a series of power management methods have been proposed [16,17,18]. Thus, the TENG can provide sustainable energy for the low power consumption embedded devices [19,20,21]. Meanwhile, the TENG can also be used as an active sensor to monitor the state of the surrounding environment, like wind speed and vibration amplitude and acceleration [22,23,24,25]. 

As we know, motor vehicles, aircraft, machine tools, and other mechanical equipment inevitably produce mechanical vibration, which can reveal the working state of the machine [26,27]. Monitoring and recording abnormal vibration of the machine is of great significance to machine safety. In general, real-time monitoring and data uploading of equipment vibration can be achieved by an accelerometer [28]. Several groups have reported that the self-powered accelerometers can be used to monitor the real-time acceleration of the equipment [29,30,31,32]. However, long-term and real-time monitoring of equipment may not be necessary in some cases. Anomalies are occasional during the equipment operation, so monitoring for a long time will cause a waste of energy. Therefore, data uploading and warning only in abnormal states is a better choice, which could greatly improve the efficiency of energy utilization. Wake-up systems have been reported that can use active sensors as triggers to wake up the system only in case of system abnormalities [33,34]. However, the systems still need an external power source, which limits their further applications. Using vibrational TENG simultaneously as a trigger of the wake-up system and as an energy harvester is expected to realize a fully self-powered vibrational wake-up system with ultra-low static energy consumption and autonomous vibration monitoring. 

Here, a self-powered and autonomous vibrational wake-up system (SAVWS) based on triboelectric nanogenerators and a micro-electromechanical system (MEMS) switch is proposed. The energy triboelectric nanogenerator (E-TENG) can harvest vibration energy to drive the wireless transmitter through a MEMS switch. The signal triboelectric nanogenerator (S-TENG) can be used as a self-powered accelerometer to control the state of the MEMS switch, which also shows good linearity in the acceleration range of 1–4.5 m/s^2^ at 30 Hz with a sensitivity of about 14.6 V/(m/s^2^). When the acceleration increases, the S-TENG will turn on the MEMS switch, and the wireless transmitter transmits an alarm signal by the energy from E-TENG with only 0.64 mJ. Using TENGs as an energy source and a sensor, the SAVWS provides a self-powered vibration monitoring solution for unattended environments, and shows extensive applications and great promise in smart factories, autonomous driving, and the Internet of Things.

## 2. Result and Discussion

### 2.1. System Framework and Working Mechanism of TENG

**Figure 1:**
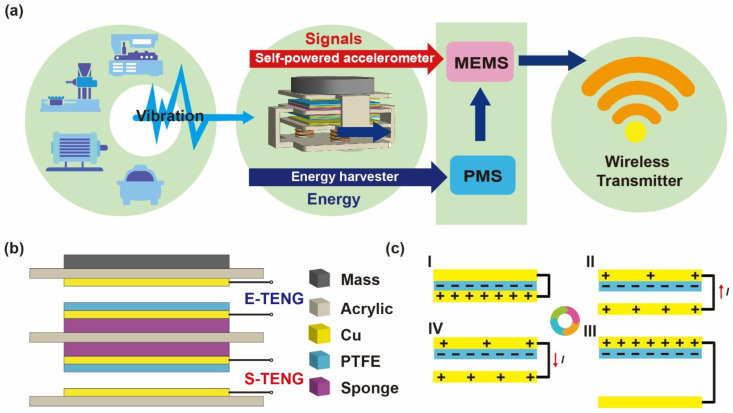
Self-powered and autonomous vibrational wake-up system (SAVWS). (**a**) System diagram of the SAVWS. (**b**) Schematic diagram of the integrated vibration TENG. (**c**) The working mechanism of the TENG in vertical contact-separation mode. (**I**) Close contact. (**II**) Begin to separate. (**III**) Stop separation. (**IV**) Begin to contact.

The working framework of the SAVWS is shown in Figure 1a, which was composed of an integrated TENG, a MEMS switch, a power management system (PMS), and a wireless transmitter. The integrated TENG could harvest the vibrational energy and measure the vibration acceleration simultaneously, which consists of E-TENG, S-TENG, mass, and four supporting springs (Figure S1a). Therein, the E-TENG could transform vibrational energy into electrical energy with an alternating current (AC) output. Then, the PMS connected to the E-TENG could convert the AC into direct current (DC) which was supplied to the transmitter module. For vibration sensing, the S-TENG could convert the vibration information into electrical signals and control the operating state of the MEMS switch. When the S-TENG detected that the acceleration of vibration exceeds a specific threshold, the MEMS switch would turn on to wake up the transmitter module to send alarm signals. What is more, the acceleration threshold could be adjusted by regulating the output characteristics of S-TENG. Finally, the alarm signals could be analyzed and recognized by the remote monitoring. As a result, a fully self-powered remote acceleration monitoring has been realized.

As shown in Figure 1b, the E-TENG and S-TENG were both in the vertical contact-separation mode. Polytetrafluoroethylene (PTFE) and Cu were chosen as triboelectric materials of TENGs due to their excellent contact electrification properties [35,36]. Owing to the excellent processability of the acrylic sheet, which was used as the base and support layer, the sponge between the Cu film and the acrylic sheet could increase the effective contact area between the triboelectric pairs. Figure 1c illustrates the working mechanism of the vertical contact-separation TENG, which is the coupling effect of contact electrification and electrostatic induction [37]. When the TENG was actuated by external mechanical vibration, the triboelectric layers began to contact and separate back and forth. Based on the triboelectric series [36], the electrons could transfer from the Cu film to the PTFE film, while they would obtain the same number of opposite charges. There was almost no electric potential difference between the two electrodes (Figure 1c(I)). Once the two films are separated, an electric potential difference would be generated between the two electrodes and drive electrons from Cu electrode to Cu film (Figure 1c(II)) until the distance between the PTFE film and Cu film reached the maximum (Figure 1c(III)). Then, the two triboelectric layers came close again. The electric potential difference between the Cu film and Cu electrode decreased gradually, and the electrons would be driven from Cu film to Cu electrode. (Figure 1c(IV)). Through contact–separation circulation, the electric potential difference constantly drove electrons to move between the Cu film and Cu electrode. In this way, the conversion of mechanical energy to electrical energy could be realized. The contact area size of TENG is shown in Figure S1b.

### 2.2. Output Characteristics of E-TENG and S-TENG

**Figure 2:**
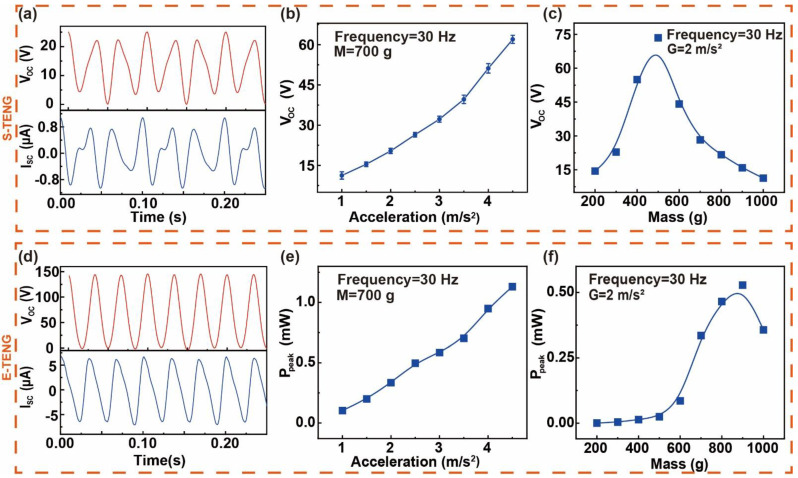
Output characteristics of S-TENG and E-TENG. (**a**) Open-circuit voltage and short-circuit current of S-TENG at the acceleration of 2 m/s^2^, 30 Hz. The open-circuit voltage output of S-TENG at different (**b**) accelerations and (**c**) mass. (**d**) Open-circuit voltage and short-circuit current of E-TENG at the acceleration of 2 m/s^2^, 30 Hz. The output peak power of E-TENG at different (**e**) accelerations and (**f**) mass.

The output characteristics of S-TENG and E-TENG have been studied, as shown in Figure 2. Figure 2a shows the open-circuit voltage (V_OC_) and short-circuit current of S-TENG under the acceleration of 2 m/s^2^, the mass of 700 g, and the frequency of 30 Hz. It can be seen that the S-TENG had stable output waveforms. The V_OC_ of S-TENG with fixed mass was tested at different accelerations. The data were from the mean of five sets of experimental data, and the error bar was taken from the standard deviation, as shown in Figure 2b. With a fixed frequency, the V_OC_ of S-TENG had good linearity in the acceleration range of 1–4.5 m/s^2^, and a sensitivity of about 14.6 V/(m/s^2^). In addition, eight masses were selected between 200 g and 1000 g to further measure the output of S-TENG, as shown in Figure 2c. The results show that the output of S-TENG would quickly increase and then decrease with the increase in mass.

The output peak power (P_peak_) of E-TENG was also measured. When the acceleration of the vibration was 2 m/s^2^, the frequency was 30 Hz and the mass was 700 g, the E-TENG had stable output waveforms as shown in Figure 2d. Figure 2e displayed the P_peak_ of E-TENG under different accelerations, and results show that the larger the acceleration, the higher the P_peak_. In addition, the P_peak_ of E-TENG with different mass is shown in Figure 2f, and it can be seen the E-TENG had a good output performance after the mass was over 500 g. Since the S-TENG and the E-TENG were integrated, changing the mass would affect both output characteristics of S-TENG and E-TENG. Therefore, the variation range of the mass was fixed between 500 g and 1000 g to ensure that the system could work stably. Besides, the V_OC_ waveforms of E-TENG and S-TENG are shown in Figure S2. The effect of frequency on the output characteristics of S-TENG and E-TENG was also measured (Figure S3). At a constant acceleration, the output performances of S-TENG and E-TENG improved as the frequency decreased. The durability of S-TENG and E-TENG were also tested. After 90,000 times of contact–separation circulation, the E-TENG and the S-TENG still had stable output performances, as shown in Figure S4. 

### 2.3. Working Principle and Performance of SAVWS

**Figure 3:**
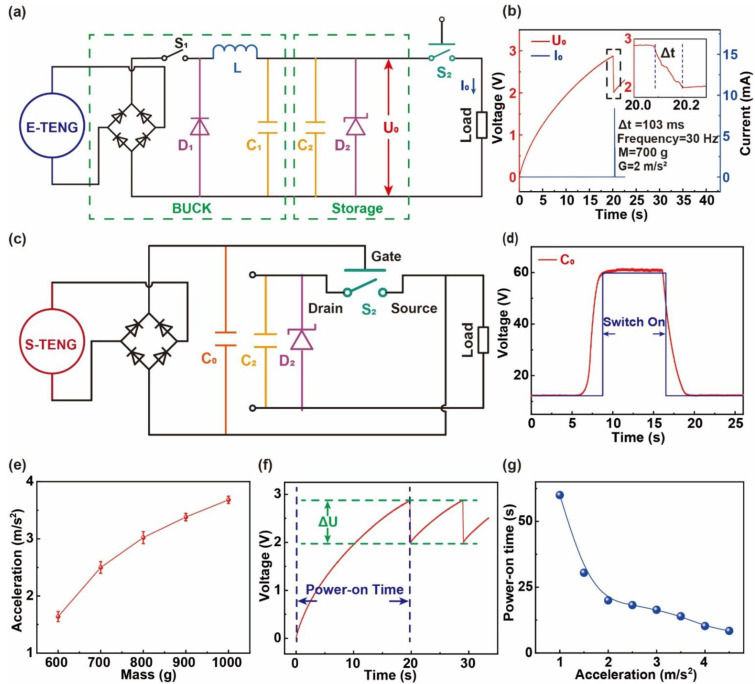
Working principle and performance of SAVWS. (**a**) The circuit schematic diagram of the E-TENG and PMS. (**b**) The voltage waveform of C_2_ and the waveform of I_0_. (**c**) The circuit schematic diagram of the control circuit of the MEMS switch. (**d**) The voltage waveform of the C_0_. (**e**) The change in acceleration threshold at different mass. (**f**) The voltage change in the transmitter module when it works, and the power-on time. (**g**) The change in power-on time under different accelerations.

Figure 3a,c shows the circuit diagrams of the SAVWS. The load and S_2_ were the wireless transmitter module and the MEMS switch, respectively. The PMS consisted of a BUCK circuit and an energy storage circuit. Therein, the BUCK circuit was composed of a rectifier bridge, series switch S_1_, diode D_1_, inductor L, and capacitor C_1_. In addition, to better understand the workflow of SAVWS, the energy flow in this system is described below. The rectifier bridge converted the AC generated by E-TENG into DC, and the voltage signals from the rectifier bridge could control the working order of the S_1_. When S_1_ was turned on, the energy from E-TENG would be temporarily stored in L. After a moment, S_1_ would turn off and the energy in L would be stored in C_1_ through the loop of L, D_1_, and C_1_. Through this process, the energy produced by E-TENG would eventually be stored in the capacitor C_2_ with high efficiency [16]. The Zener diode (D_2_) was added to protect C_2_ from the damage of high voltage. Figure S5 shows a comparison of capacitor charging efficiency between the direct charge circuit with only one rectifier bridge and the PMS circuit. Figure 3b shows the waveforms of the U_0_ and the I_0_. The U_0_ was the voltage of the C_2_, which could demonstrate the working state of the transmitter module, and the I_0_ was the current flowing through the transmitter. When the E-TENG continuously harvested vibrational energy and S_2_ was in the closed state, the max value of I_0_ and U_0_ were 8.38 mA and 2.9 V, respectively. The time for the value of U_0_ dropping from 2.9 V to 2 V, which was 103 ms, indicated the working time of the transmitter module. The maximum current of 8.38 mA and the extremely short working times demonstrated the low power consumption of the transmitter.

The MEMS switch used in the system was voltage-sensitive and controlled by the output voltage of S-TENG, as shown in Figure 3c. Therein, the C_0_ was connected at the gate and at the source of the MEMS switch to stabilize the MEMS state. When the voltage of C_0_ reached the threshold, the drain and the source would be in a short-circuit state. The internal structure and microstructural diagram of the MEMS switch were shown in Figure S6. The relationship between the state of S_2_ and the voltage of C_0_ is shown in Figure 3d. The output voltage of the S-TENG with the mass of 700 g would turn on the MEMS switch under the vibration of acceleration of 2.5 m/s^2^. When the vibration occurred, the voltage of C_0_ would be raised to 60 V within 2.72 s and S_2_ would turn on. After the vibration stopped, the voltage of C_0_ would drop rapidly below 48 V within 450 ms and S_2_ would turn off. It indicated that the voltage change in C_0_ was not instantaneous, and there was a time delay between the state of S_2_ and the voltage of C_0_. What is more, the working time of the transmitter module was 103 ms. Thus, the response time of the system to abnormal acceleration was 2.82 s. 

The detailed workflow of the SAVWS is as follows. As the integrated vibrational TENG worked, the E-TENG and the S-TENG would start harvesting energy and monitoring the acceleration of vibration, respectively. When the acceleration of the vibration exceeded a certain value, the S-TENG would turn on the MEMS switch and wake up the transmitter module to send alarms. In addition, the acceleration value of turning on the MEMS switch, which was called the acceleration threshold, could change by the mass. Therefore, to realize the monitoring of various accelerations, the relationship between acceleration threshold and mass was studied. As shown in Figure 3e, the acceleration threshold increased with the raising of the mass. The data in Figure 3e were also from the mean of five sets of experimental data, and the error bar was taken from the standard deviation. It can be seen that the SAVWS could monitor the accelerations from 1.6 m/s^2^ to 3.7 m/s^2^ by changing the mass. 

The load was a transmitter module, which was composed of a voltage monitoring circuit and a wireless transmitter. The circuit diagram of the transmitter module is shown in Figure S7. The voltage monitoring circuit could be regarded as an electronic switch, which could monitor the voltage of C_2_ when the module was woken up by the MEMS switch. When the voltage of C_2_ exceeded 2.9 V, the wireless transmitter would start working. After the voltage of C_2_ fell below 2 V, the voltage monitoring circuit would turn off the wireless transmitter. This process could prevent the voltage of C_2_ from being too low to affect the operation of the transmitter. As shown in Figure 3f, when the transmitter module sent out an alarm signal, the voltage of C_2_ dropped from 2.9 V to 2 V. Because the capacitance C_2_ was 330 μF, it could be concluded that only 0.64 mJ energy was needed for the wireless transmitting module to send an alarm signal. Because of the protection of the voltage monitoring circuit, the voltage of C_2_ should be at least 2.9 V before the system could start sending alarm signals. The time that C_2_ took to increase from 0 to 2.9 V was called the power-on time. Furthermore, the change in power-on time under different accelerations was shown in Figure 3g. The power-on time decreased from 60 s to 8.4 s as the acceleration increased from 1 m/s^2^ to 4.5 m/s^2^. Since the abnormal acceleration in a machine is an occasional event, the maximum power-on time of 60 s could fully meet the performance requirements of the system. 

### 2.4. Working Principle and Performance of SAVWS

**Figure 4:**
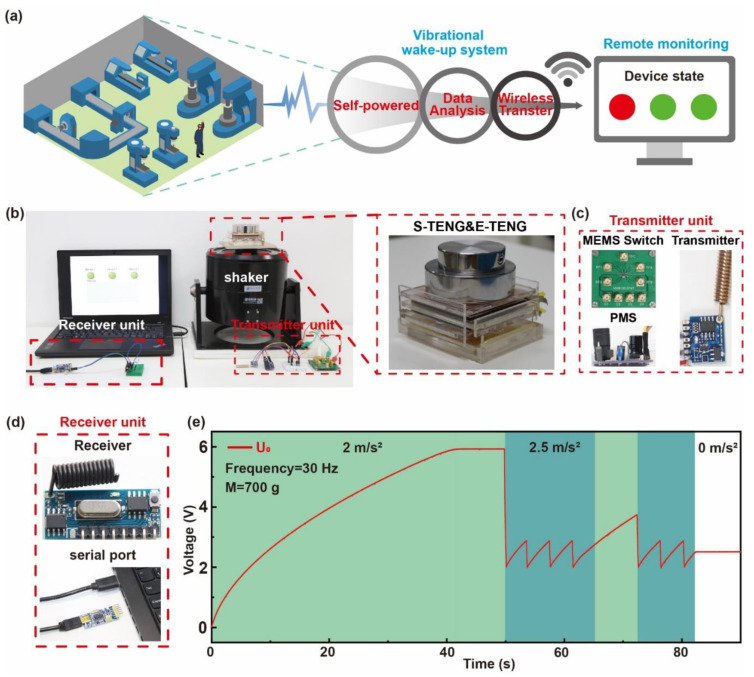
Application of the SAVWS for the sustainable and autonomous wireless monitoring system. (**a**) The diagram of the SAVWS for a wireless monitoring system. (**b**) Experimental environment and photos of SAVWS components. (**c**) Photos of the transmitter unit. (**d**) Photos of the receiver unit. (**e**) The stored and regulated voltage waveforms in the working processing.

The SAVWS can be installed in mechanical equipment and use the energy harvested from the vibration of the machines to monitor the working state of machines (Figure 4a). When the acceleration of the machine is abnormal, the SAVWS sends alarm signals, and the receiver transfers the alarm to the computer through the serial port. To further explore the working process of SAVWS, the working environment of the SAVWS was simulated in the laboratory. A shaker was used to provide vibration with a frequency of 30 Hz to simulate the vibration of machines during the experiment. The mass was 700 g, which meant the acceleration threshold of the system was about 2.5 m/s^2^. The experimental environment is shown in Figure 4b. The photos of the transmitter unit and receiver unit are shown in Figure 4c,d.

During the working process, the voltage waveform of the C_2_ indicated the working state of SAVWS (Figure 4e). In the initial state, the E-TENG had no output and the voltage of C_2_ was zero. When the acceleration arrived at 2 m/s^2^, the E-TENG started charging the capacitor C_2_ through the PMS. Due to the protection of the Zener diode (D_2)_, the voltage of C_2_ would stop increasing when the voltage was about to 6 V, and then the acceleration was set to 2.5 m/s^2^ to simulate the abnormal acceleration of the machine. Under this circumstance, the S-TENG could detect that acceleration had exceeded the threshold and controlled the MEMS switch to wake up the transmitter module. The energy in the C_2_ was supplied to the transmitter module to continuously send alarm signals. Due to the voltage monitoring circuit, the transmitter module stopped working as the voltage of C_2_ was lower than 2 V until it increased to 2.9 V. In this process, the remote monitoring could receive alarm signals periodically. The acceleration was then reduced to 2 m/s^2^, and the MEMS switch turned off the transmitter module. Meanwhile, the voltage of C_2_ continued to increase until the next abnormal acceleration came. After a while without receiving alarm signals, the remote monitoring would clean the warning. When the abnormal acceleration came again, the system would send alarm signals once more. In the experimental environment, the maximum transmission distance of the transmitter module was verified to be about 50 m. At last, due to the extremely low leakage current of the MEMS switch, the voltage of C_2_ hardly changed after the vibration stopped. Figure S8 demonstrates the ultra-low leakage current of the MEMS switch at different gate-to-source voltages. Video S1 demonstrates the demo of the system. By continuously harvesting vibrational energy in the environment, the SAVWS could enable fully self-powered and continuous acceleration monitoring and alarm, which has wide applications in environmental monitoring of unattended factories.

## 3. Materials and Methods

### 3.1. The Materials of Vibrational TENGs

Acrylic sheets with a thickness of 2.5 mm were used as support material. The springs (0.7 mm in diameter and 10 mm in length) were selected as the support. The thickness of the PTFE film as the triboelectric layer was 0.08 mm and the thickness of the Cu film was 0.05 mm. The thickness of the sponge layer was 3 mm.

### 3.2. The Fabrication of Integrated TENG

First, a 90 mm long and 68 mm wide acrylic sheet was used as a base plate. Then, four springs were located at the four corners of the base plate, which supported another acrylic sheet as a moving layer. The springs were fixed to the acrylic sheets with hot melt glue. The Cu film was attached to the upper and lower layers of the moving layer as the electrode and triboelectric layer. An 80 mm long and 68 mm wide acrylic sheet was used as a stationary layer which was supported by the base plate. Next, the front and back sides of the fixed layer were attached with a sponge, Cu film, and PTFE.

The contact area of the triboelectric layers was 62 mm × 62 mm. The weight (60 mm in diameter) was used as the mass. The integrated vibrational TENG had a height of 45 mm, a width of 68 mm, and a length of 90 mm. The optical picture of the specific internal structure of the integrated TENG is shown in Figure S9.

### 3.3. Measurement and Electronic Components

A shaker (KSI-758ST500) with a control system (VT-9002) was used to simulate the mechanical vibration. The voltage signals in the experiment were measured by an electrometer (Keithley 6514 system electrometer). During the experiment, a shaking table was used to provide vertical vibrations, and the open-circuit voltage and short-circuit current were measured by the electrometer connected to the poles of the TENGs. The voltage of capacitors was measured by the electrometer connected to both ends of them. 

The models of electronic components used in the system circuit are as follows: rectifier bridge: DB107; D_1_: MUR460; C0: 470 pF, Ceramics capacitor; C_1_: 2200 pF, Ceramics capacitor; C_2_: 330 μF, Aluminum electrolytic capacitor; D_2_: 6 V; Zener diode. The model of the transmitter was DTX1K, and the manufacturer was IoT Electronics. The model of the MEMS switch was MM5130 manufactured by Menlomicro. The display interface of remote monitoring was made by a commercial software LabVIEW.

## 4. Conclusions

In summary, a self-powered and autonomous vibrational wake-up system based on the TENGs and MEMS switch was proposed. The E-TENG had been experimentally proven to have outstanding characteristics, which can provide sustainable and stable energy to the system in a continuous vibration environment. The S-TENG had also been demonstrated to have good linearity in the acceleration range of 1–4.5 m/s^2^ at 30 Hz with a sensitivity of about 14.6 V/(m/s^2^). The SAVWS realized the energy harvesting and vibration monitoring by integrating the E-TENG and the S-TENG. Through the MEMS switch, the SAVWS could monitor an acceleration threshold in only 2.82 s, which could be adjusted by changing the mass. With a low-powered design, the wireless transmitter could transmit an alarm signal by the energy from the E-TENG with only 0.64 mJ. As a result, the system could realize the efficient utilization of the energy generated by the E-TENG and fast response to abnormal acceleration. Using the TENG simultaneously as an energy source and a sensor, the SAVWS provided a self-powered vibration monitoring solution for unattended environments, and showed extensive applications and great promise in smart factories, autonomous driving, and the Internet of Things. 

## Data Availability

Not applicable.

## Supplementary Materials

The following supporting information can be downloaded at: https://www.mdpi.com/article/10.3390/s22103752/s1, Figure S1: The structure schematic of the integrated vibration TENG; Figure S2: Characteristics of the S-TENG and E-TENG; Figure S3: The open-circuit voltage of S-TENG and E-TENG in different frequencies, Figure S4: The durability test results of the S-TENG and E-TENG, Figure S5: Comparison of direct charge circuit with only one rectifier bridge and through the PMS charging for a 330 μF capacitance, Figure S6: Structure and microstructural diagram of MEMS switch, Figure S7: The circuit schematic diagram of the load, Figure S8: Leakage current of MEMS switch, Figure S9: The optical picture of the specific internal structure of the integrated TENG, Video S1: The working demo of SAVWS.
