# Supplementary material for: Self-Powered and Autonomous Vibrational Wake-Up System Based on Triboelectric Nanogenerators and MEMS Switch
Source: Sensors (Basel). 2022 May 14;22(10):3752. doi: 10.3390/s22103752 (PMC9145876; doi:10.3390/s22103752)
Supplement: Supplementary file 1 [file sensors-22-03752-s001.zip › sensors-1679485-supplementary.pdf]

sensors

## Supplementary Materials

# Self-Powered and Autonomous Vibrational Wake-Up System Based on Triboelectric Nanogenerators and MEMS Switch

**Yuan Lin [1,2], Youchao Qi [2,3], Jiaqi Wang [2], Guoxu Liu [2,3], Zhaozheng Wang [2,3], Junqing Zhao [2,3], Yi Lv [2,3], Zhi Zhang [2,3], Ning Tian [4], Mengbi Wang [5], Yuanfen Chen [1,6,*] and Chi Zhang [1,2,3,6,*]**

[1] School of Mechanical Engineering, Guangxi University, Nanning 530004, China; 1911301021@st.gxu.edu.cn
[2] CAS Center for Excellence in Nanoscience, Beijing Key Laboratory of Micro-Nano Energy and Sensor, Beijing Institute of Nanoenergy and Nanosystems, Chinese Academy of Sciences, Beijing 100083, China; qiyouchaoo@163.com (Y.Q.); 202010410039@imut.edu.cn (J.W.); liuguoxu@binn.cas.cn (G.L.); wangzhaozheng@binn.cas.cn (Z.W.); zhaojunqing@binn.cas.cn (J.Z.); lvyi@binn.cas.cn (Y.L.); zhangzhi@binn.cas.cn (Z.Z.)
[3] School of Nanoscience and Technology, University of Chinese Academy of Sciences, Beijing 100049, China
[4] Tsinghua Innovation Center in Zhuhai, Zhuhai 519080, China; tianning2001@vip.sina.com
[5] State Key Laboratory of Precision Measurement Technology and Instruments Department of Precision Instrument Tsinghua University, Beijing 100084, China; wmb@mail.tsinghua.edu.cn
[6] Center on Nanoenergy Research, School of Physical Science and Technology, Guangxi University, Nanning 530004, China
* Correspondence: yuanfenchen@gxu.edu.cn (Y.C.); czhang@binn.cas.cn (C.Z.)

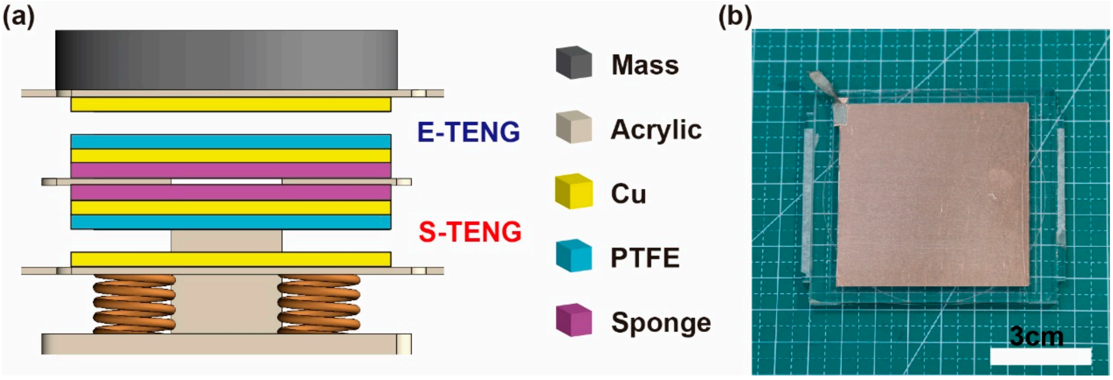


**Figure S1. The structure schematic of the integrated vibration TENG.** (**a**) The 3D structure schematic of the integrated vibration TENG. (**b**) The photograph of the actual contact area for TENG.

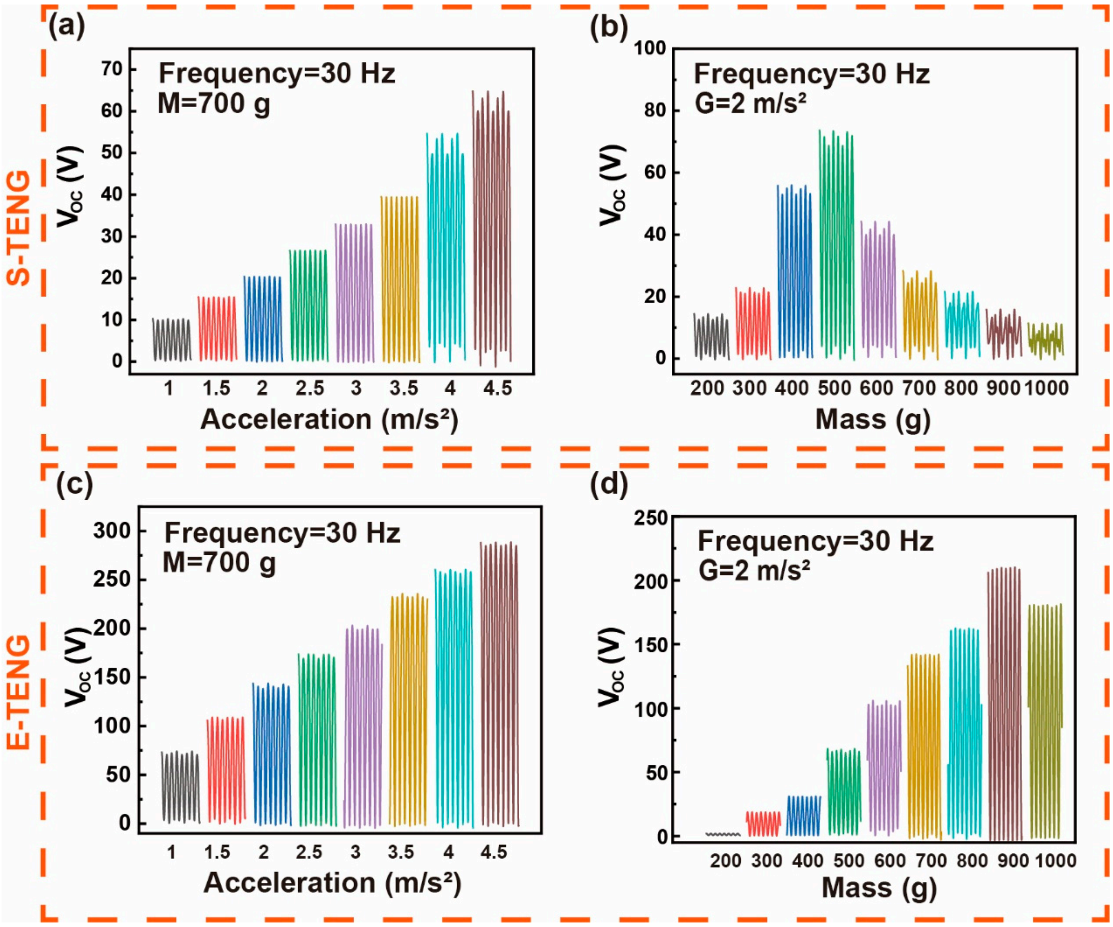


**Figure S2. Characteristics of the S-TENG and E-TENG.** (**a**) (**b**) The open-circuit voltage waveforms of the S-TENG at different accelerations and mass. (**c**) (**d**) The open-circuit voltage waveforms of the E-TENG at different accelerations and mass.

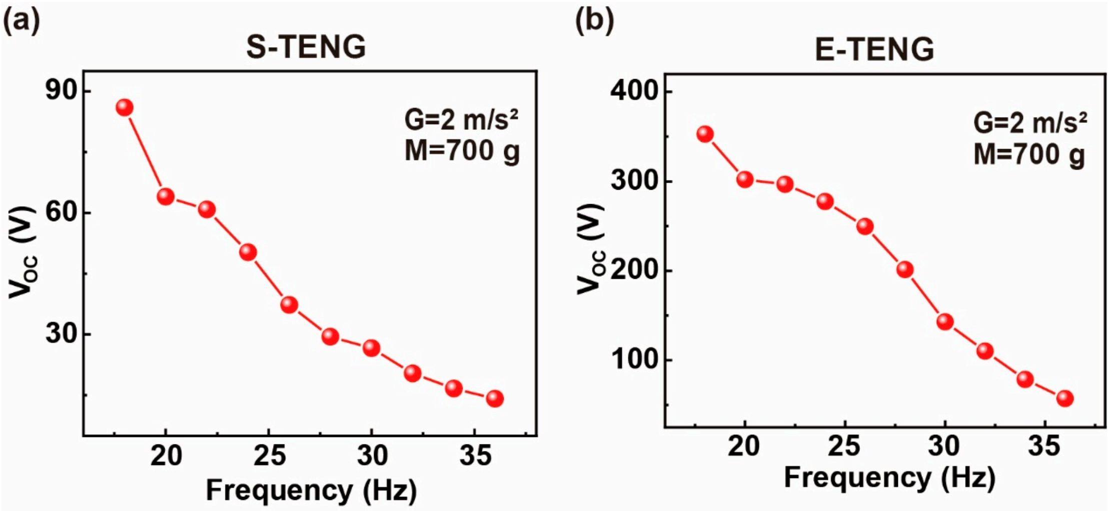

**Figure S3.** The open-circuit voltage of S-TENG and E-TENG in different frequencies.

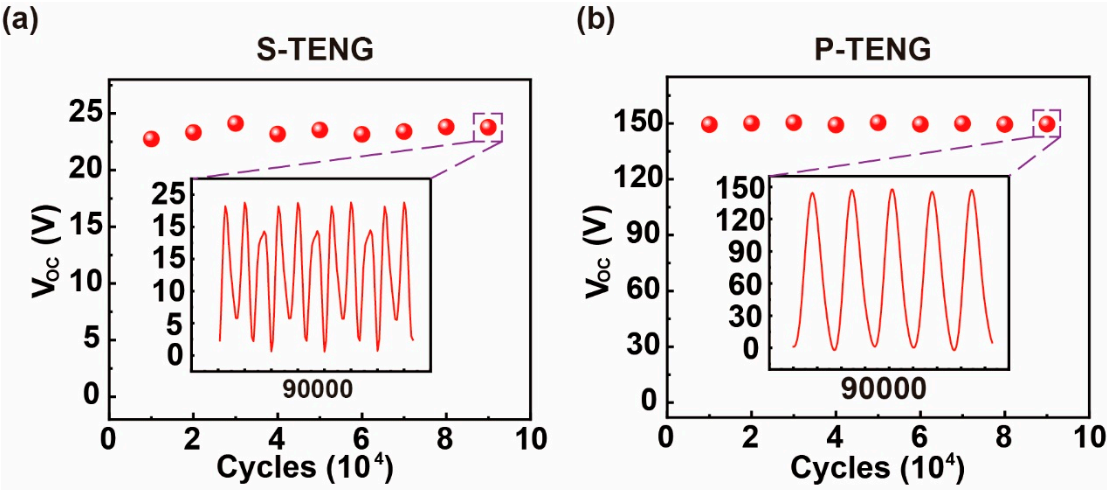

**Figure S4.** The durability test results of the S-TENG and E-TENG.

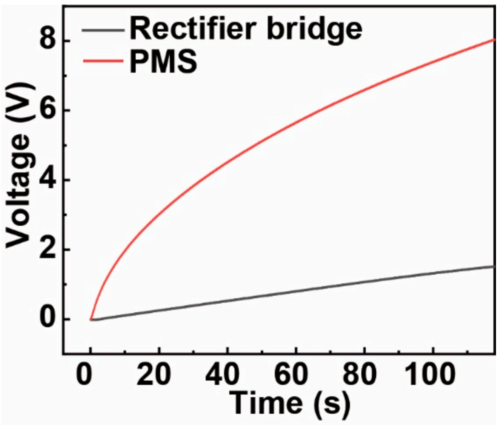

**Figure S5.** Comparison of direct charge circuit with only one rectifier bridge and through the PMS charging for a 330μF capacitance.

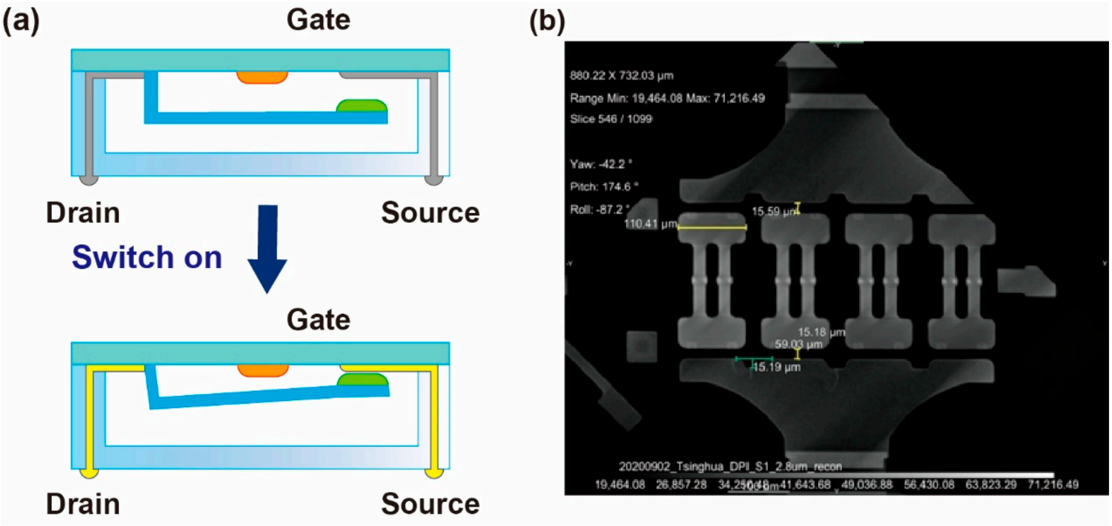


**Figure S6.** Structure and microstructural diagram of MEMS switch.

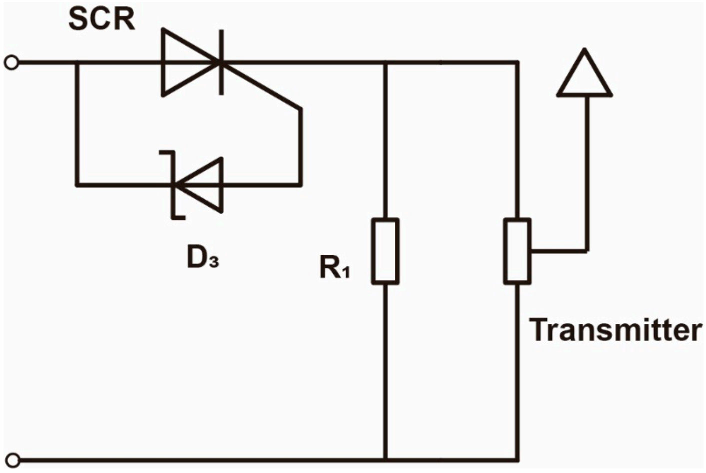


**Figure S7** The circuit schematic diagram of the load. The SCR model is EC103M1. The $D_3$ is a Zener diode with a value of 3.3V. The $R_1$ has a resistance of 4.7kΩ, which acts as a discharge resistor to prevent the SCR from closing until the signal has been sent completely.

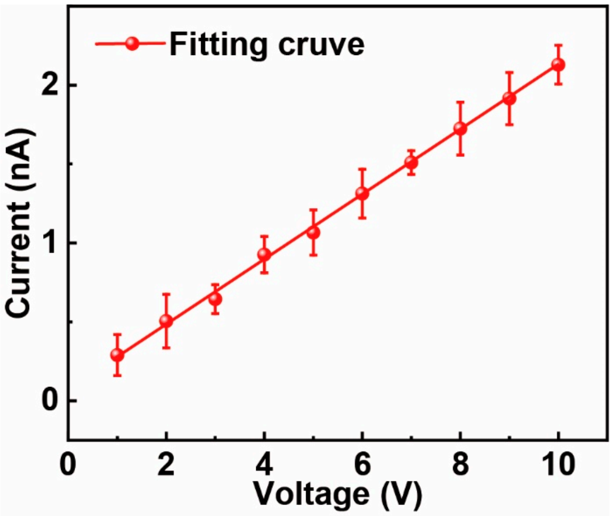


**Figure S8. Leakage current of MEMS switch.** The leakage current increases linearly as the voltage between beam and contact increasing.

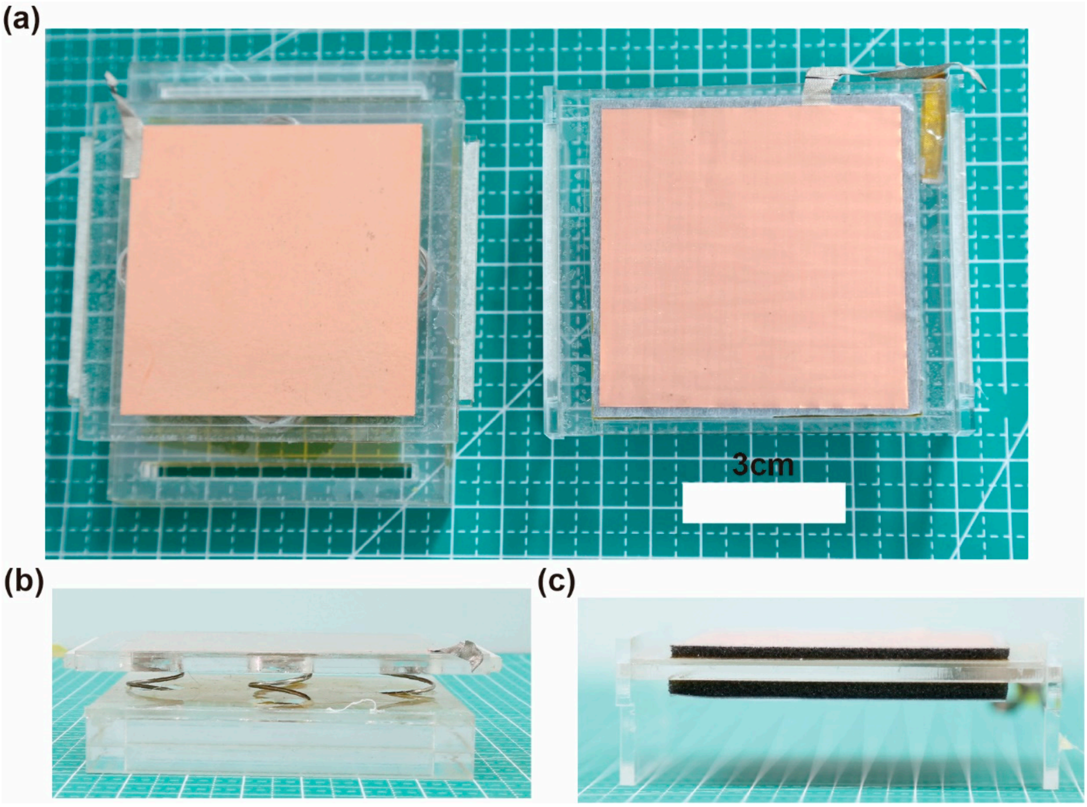


**Figure S9. The optical picture of the specific internal structure of the integrated TENG.** (**a**) The photo of the base plate, moving layer, and stationary layer. (**b**) Side view of the base plate and the moving layer. (**c**) Side view of the stationary layer.

**Supplementary Videos:**

**Video S1.** The working demo of SAVWS.
